# Ecology and social system of northern gibbons living in cold seasonal forests

**DOI:** 10.24272/j.issn.2095-8137.2018.045

**Published:** 2018-05-12

**Authors:** Zhen-Hua Guan, Chang-Yong Ma, Han-Lan Fei, Bei Huang, Wen-He Ning, Qing-Yong Ni, Xue-Long Jiang, Peng-Fei Fan

**Affiliations:** 1 Yunnan Academy of Biodiversity, Forest Disaster Warning and Control Key Laboratory, Southwest Forestry University, Kunming Yunnan 650224, China; 2 School of Life Sciences, Sun Yat-Sen University, Guangzhou Guangdong 510275, China; 3 State Key Laboratory of Genetic Resources and Evolution, Kunming Institute of Zoology, Chinese Academy of Sciences, Kunming Yunnan 650223, China; 4 College of Animal Sciences and Technology, Sichuan Agricultural University, Chengdu Sichuan 611130, China

**Keywords:** Northern gibbon, Ecology, Social system, Adaptation, Group size

## Abstract

Gibbons in China represent the northernmost margin of present day gibbon species distribution (around N25${}^{\circ }$). Compared to tropical habitats, northern gibbon habitats are characterized by low temperatures and remarkable seasonal variation in fruit abundance. How gibbons adapt to their cold and seasonal habitats and what ecological factors affect their sociality are key questions for understanding their ecology and social system evolution, the elucidation of which will contribute to the conservation of these special populations/species. According to preliminary short-term studies, northern gibbons consume more leaves and use larger home ranges than tropical gibbons. Interestingly, some *Nomascus* groups consist of more than one adult female. However, these preliminary results are not well understood or incorporated into current socio-ecological theories regarding gibbon species. To better understand northern gibbons, our team has systematically studied three habituated groups of *Nomascus concolor*, three groups of *N. nasutus*, and two habituated groups of *Hoolock tianxing* since 2002. In this paper, we stress the challenges facing gibbons living in northern habitats and summarize their behavioral adaptations to their harsh environments. We also describe the northern gibbon social system and discuss the potential relationships between their ecology and sociality. Finally, we highlight future research questions related to northern gibbons in China.

## INTRODUCTION

Gibbons (Hylobatidae) are small apes that live in tropical and subtropical forests in southeastern Asia (Bartlett, 2011). These apes are characterized by several interrelated features, including long arms for brachiation, terminal branch feeding, frugivory, pair-living, and territoriality (Brockelman, 2009). In tropical forests, gibbons rely heavily on ripe fruit pulp (Chivers, 1984), with fruits including figs comprising >60% of their annual diet, though siamang (*Symphalangus syndactylus*) is more folivorous (reviewed in Bartlett, 2011). Fruit is often distributed in small and defensible patches, resulting in food competition among females. Female gibbons are considered intolerant to each other and live solitarily (Bartlett, 2011; Kappeler et al., 2013; Leighton, 1987; Wrangham, 1987), with males unable to successfully defend ranges occupied by two or more females (Bartlett, 2009). Males may thus benefit from pair-living through 1) infanticide defense against non-resident, unmated males (Borries et al., 2011; Opie et al., 2013; van Schaik & Kappeler, 2003); 2) increased paternal certainty by mate guarding (Palombit, 1996, 1999); 3) limited opportunities for the female to evaluate potential sexual partners (Palombit, 1996, 1999); and 4) enhanced foraging efficiency by sharing knowledge with females (Brockelman, 2009) or by ensuring that resources are not unknowingly depleted by foraging apart (Terborgh, 1983). Females benefit from pair-living through food and territory defense, direct infant care provided by males (reported in siamangs, Lappan, 2008), and enhanced foraging efficiency (Brockelman, 2009). The mated pair use a small home range of on average 42 hm^2^, and actively defend 82% of the area (Bartlett, 2011) through direct intergroup conflicts (Bartlett, 2003) and regularly occurring loud morning songs (Geissmann, 2002; Mitani, 1985). Both sexes benefit from knowledge of this intensively-used territory and goal-directed foraging strategies (Brockelman, 2009). This, in turn, reinforces territory defense and small home range, and consequently often limits gibbon group size (Brockelman, 2009; Brockelman et al., 2014). Bartlett (2009) first integrated seasonality into territory defense. He argued that stable food abundance is a prerequisite for territory defense. However, most information on gibbons comes from only a few studied species (mainly *Hylobates lar* and *S. syndactylus*) living in lowland tropical forests (Bartlett, 2009, 2011; Brockelman et al., 1998; Chivers, 1974; Lappan, 2008; Palombit, 1996, 1999).

**Figure 1:**
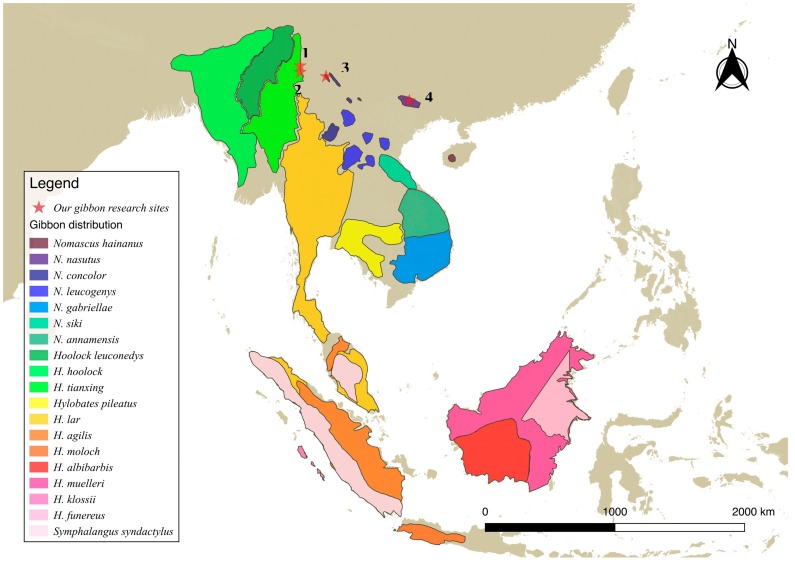
Distribution of the gibbon species: *Nomascus* (7 species); *Hoolock* (3 species); *Hylobates* (9 species); *Symphalangus* (1 species) (According to Thinh et al., 2010a, 2010b; Fan et al., 2017; IUCN, 2017)

Historically, gibbons were distributed widely in China. As recently as 500 years ago, their melodic songs could be heard along The Three Gorges in central China and even further north (close to N35${}^{\circ }$) (Turvey et al., 2015; Zhou & Zhang, 2013). Although the known gibbon range has shrunk considerably from north to south, and is now restricted to Yunnan, Guangxi, and Hainan in southern China (Fan, 2017; Turvey et al., 2015; Zhou & Zhang, 2013), these sites still represent the northernmost margin of present day gibbon distribution (around N25${}^{\circ }$, e.g., *N. concolor* in Wuliang Mountain: Jiang et al., 2006; *H. tianxing* in Gaoligongshan: Fan et al., 2011b, 2017), second only to the *Hoolock* gibbons in India and Myanmar (Groves, 1967). In this paper, we arbitrarily refer to the populations of *N. concolor*, *N. leucogenys*, *N. hainanus*, *N. nasutus*, and *H. tianxing* in China as “northern” gibbons (Figure 1). It does not mean that these gibbons are distributed in northern areas but is used for comparison between these gibbon populations and gibbons living in “southern” tropical forests.

Our gibbon research sites are also marked on the map.

Based on short-term studies of unhabituated northern gibbons in China, early researchers reported on the differences in ecology and behavior compared to tropical gibbons. For example, northern gibbons were less frugivorous (*N. leucogenys*: Hu et al., 1990; *N. concolor*: Chen, 1995; Lan, 1993), occupied larger home ranges (*N. hainanus*: Liu et al., 1989; *N. leucogenys*: Hu et al., 1990; *N. concolor*: Jiang et al., 1994a; Jiang & Wang, 1999), and some groups included more than one adult female (*N. concolor*: Haimoff et al., 1986, 1987; *N. hainanus*: Liu et al., 1989; Jiang et al., 1994a, 1994b; Jiang & Wang, 1999). Although early studies reported obvious differences, detailed observations on these gibbons were hampered in China by a lack of habituated wild gibbons due to intense human hunting pressure and difficult terrain (Yin et al., 2016). Systematic, longitudinal research became possible only recently after several *N. concolor* and *H. tianxing* groups became habituated to observers in Mt. Wuliang and Mt. Gaoligong (Fan & Jiang, 2008; Fan et al., 2008, 2009a, 2013; Yin et al., 2016). In 2006, *N. nasutus* was rediscovered in a small karst forest patch in Bangliang, Jingxi County, Guangxi (Chan et al., 2008). Researchers could observe these gibbons from fixed observation posts located on hill tops or forest gaps due to the steep karst terrain and low canopy height (Fan et al., 2011a, 2012). With accumulating data from different species living in different habitats, the picture of northern gibbon ecology and sociality, although still incomplete, is becoming clearer.

## CHALLENGES FACING NORTHERN GIBBONS IN CHINA

The human population in China has increased rapidly and currently stands at over 1.3 billion (National Bureau of Statistics of China, http://www.stats.gov.cn/english/). The resulting increase in demand for cropland, together with the steel-making campaign of 1958, has led to the clearance of vast tracts of forest (Jiang et al., 2006; Zhang, 2000). Deforestation and habitat fragmentation caused by expansion of crop cultivation and plantations are the main causes of gibbon decline and extinction in China (Fan et al., 2011a, 2011b, 2013, 2014; Fan, 2017; Jiang et al., 2006; Zhang et al., 2010; Zhou et al., 2005), with large-scale hunting in the 1970s and 1980s also having a considerable impact. A total of 81 365 primate skins were collected by Chinese government officials between 1974 and 1980 (Quan et al., 1981). While many skins were from macaque species (*Macaca mulatta* and *M. arctoides*), gibbons were also hunted during this period (Fan, 2017; Quan et al., 1981). In the late 1980s and early 1990s, many Chinese gibbon populations had declined to such low levels that recovery was considered unlikely. By the late 1980s, the remaining populations of three species had decreased to less than 40 individuals (*N. hainanus*: Liu et al., 1989; *H. lar*: Yang et al., 1987; *N. leucogenys*: Hu et al., 1990), and until recently *N. nasutus* was considered extinct in China (Tan, 1985). To date, the numbers of *H. lar* and *N. leucogenys* have not recovered and both species are now assumed to be ecologically extinct in China (Fan et al., 2014; Grueter et al., 2009). After more than 20 years of intensive conservation efforts, *N. hainanus* remains highly threatened with extinction (Fellowes et al., 2008; Zhou et al., 2005) and is the most endangered primate species in the world (Fan, 2017), with only one population of less than 30 individuals surviving in Bawangling. Fortunately, a small population of about 20 individuals of *N. nasutus* was rediscovered in Bangliang, Jingxi County, Guangxi in 2006 (Chan et al., 2008). The remaining gibbon populations in China survive in montane or karst forests in several isolated mountain regions that are unsuitable for cultivation.

Gibbon habitats in these northern mountain areas are much colder than tropical forests (Table 1). For example, the annual mean temperature in Dazhaizi, Mt. Wuliang is 16.6 ${}^{\circ }$C (at 2 000 m a.s.l.: Fan & Jiang, 2008) and is even colder at Nankang (13.3 ${}^{\circ }$C, at 2 200 m a.s.l.: Fan et al., 2013) and Banchang, Mt. Gaoligong (13.0 ${}^{\circ }$C, at 2 300 m a.s.l.: Fan et al., unpublished data). At these sites, temperatures can drop below zero and forests are sometimes covered in snow (Fan & Jiang, 2008; Fan et al., 2013). Thus, the resident gibbons likely face increased thermoregulatory costs compared to tropical gibbon populations. Nakayama et al. (1971) reported that the energy expenditure of outdoor-living captive Japanese macaques (*M. fuscata*) at 5.2 ${}^{\circ }$C was 2.5 times greater than that at 29.5 ${}^{\circ }$C.

**Table 1:** Behavioral ecology information of northern gibbons

Species	Location^a^	Annual rainfall (mm)	Annual mean temperature (${}^{\circ }$C)	Group size (*n* groups)	Altitude (m)	Daily pat length (m)	Home range size (hm^2^)	Frugivory (%)	Mating system^b^	Reference
2*Hoolock tianxing	1. **Banchang** Mt. Gaoligong	1 462	13.0	4.0 (*n*=4)	1 900–2 500	NA	>93	NA	M	Yin et al., 2016; Fan et al., under review
	2. **Nankang** Mt. Gaoligong	1 801	13.3	3.0 (*n*=1)	1 900–2 300	1 162±506	88	49	M	Fan et al., 2013; Zhang et al., 2014
*Nomascus concolor*	3. **Dazhaizi** Mt. Wuliang	1 607	16.6	6.6 (*n*=5)	1 900–2 700	1 391±703	>100	44	P	Jiang & Wang, 1999; Fan et al., 2006; Fan & Jiang, 2008; Fan et al., 2009a
*N. nasutus*	4. **Bangliang** Guangxi	1 565	19.6	6.6 (*n*=5)	1 804–1 363	1 268±512	130	50	P	Fan et al., 2010, 2011a, 2012, 2015

^a^: Locations are marked on Figure 1. ^b^: “P” represents polygynous and “M” represents monogamous. NA: Not available.

Fruit productivity decreases with increasing latitude and altitude (Hanya & Aiba, 2010), and northern gibbons are known to consume less fruit than tropical gibbons (reviewed in Fan et al., 2013). Fruiting seasonality also increases with latitude, and fruiting seasons are longer in the tropics and shorter toward the poles (Ting et al., 2008). At Mt. Ailao, a site supporting one of the two largest populations of *N. concolor* in China (Li et al., 2011), figs are absent and other fruit is only available for four months of the year (Chen, 1995; Sun et al., 2007). Haimoff et al. (1987) argued that Mt. Wuliang represents an extreme environment for gibbons due to the unavailability of fruit for much of the year, during which time gibbons must subsist on leaves (also see Fan et al., 2009a). In Mt. Gaoligong, Nankang, fruit availability drops close to zero between December and February (Fan et al., 2013). These findings raise the question of how northern gibbons adapt to their cold and seasonal habitats.

## ECOLOGY OF NORTHERN GIBBONS

Northern gibbons live in sizable home ranges (*N. concolor*: >100 hm^2^, *N. nasutus*: 130 hm^2^, *H. tianxing*: >93 hm^2^ in Table 1; *N. leucogenys*: 540 hm^2^, Hu et al., 1990; *N. hainanus*: 200–500 hm^2^, Liu et al., 1989, yearly 149 hm^2^ in Bryant et al., 2016), which are considerably larger than those of tropical gibbons (reviewed in Bartlett, 2011). In mountainous areas, trees are patchily distributed in different microhabitats due to variables such as soil, altitude, sunlight, water flow, and topography (Fan et al., 2011a; Sun et al., 2007; Tian et al., 2006). Tree species such as *Saurauia napaulensis* and *Ficus neriifolia* occur along streams on damp shady slopes, whereas Tetrastigma delavagi occurs on the sunny slopes at Dazhaizi, Mt. Wuliang (Tian et al., 2006). *Ficus glaberrima* is distributed on slopes and valleys, whereas *Platycarya longipes* occurs on hill tops. The karst habitat used by *N. nasutus* was classified into four different types (Fan et al., 2011a).

Patchily distributed plant species create variable food maps for northern gibbons. To obtain sufficient sustenance, the group of *N. concolor* in Mt. Wuliang (Fan & Jiang, 2008) and *H. tianxing* in Mt. Gaoligong (Zhang et al., 2014) shift their monthly home range according to the seasonal availability of food species. Although their monthly home ranges (*N. concolor*: 37±12 hm^2^; *H. tianxing*: 28±15 hm^2^) are comparable to the home ranges of tropical gibbons (avg. 34±9 hm^2^, calculated from Table 17.3 in Bartlett, 2011, excluding *Nomascus*), they occupy a larger total home range to satisfy their annual food requirements (Fan & Jiang, 2008; Zhang et al., 2014). Furthermore, they do not use their large home range evenly, but instead heavily utilize smaller areas with greater food resources (Fan & Jiang, 2008). They also do not significantly increase their daily path length compared with tropical gibbons (northern gibbons in Table 1; tropical *Hylobates* gibbons: avg. 1 300 m), except for some siamang species (e.g., *S. syndactylus*: 0.8 km, reviewed in Bartlett, 2011).

In response to the overall low availability of fruit, early researchers suggested that northern gibbons ate more leaves and less fruit than tropical gibbons (Chen, 1995; Hu et al., 1990; Lan, 1993). This hypothesis was supported by several later studies with at least one-year duration. For example, the overall diet of *N. concolor* at Dazhaizi, Mt. Wuliang, was comprised of 46.5% leaves and 44.1% fruit (Fan et al., 2009a), with similar results reported for *H. tianxing* in Nankang, Mt. Gaoligong (49.1% fruit and 43.3% leaves, Fan et al., 2013) and *N. nasutus* in Bangliang (50% fruit and 35% leaves, Fan et al., 2012). Comparatively, the diets of tropical gibbons are typically comprised of 64% fruit and 25% leaves, except for the more folivorous siamangs (49% fruit and 38% leaves) (Bartlett, 2011). Although northern gibbons consume less fruit than tropical gibbons, they prefer fruit when it is available (Fan et al., 2012). When non-fig fruit is more abundant, *H. tianxing* feed on fewer leaves (Fan et al., 2013). Although Fan et al. (2009a) did not monitor fruit availability at Dazhaizi, Mt. Wuliang, the *N. concolor* group studied spent more time feeding on fruit in March of the second year when fruit was more available (Fan et al., 2009a). When fruit is less accessible or absent, northern gibbons switch their diet to leaves and buds (Fan et al., 2009a, 2012, 2013), accounting for 83% of the *N. concolor* diet in March (Fan et al., 2009a), and increase their feeding time (Fan et al., 2008).

When leaves dominant their diets, gibbons decrease their travel time and shorten their daily path length as leaves contain fewer available calories per gram than fruit (Raemaekers, 1978) and are more abundant and evenly distributed in the forest (Fan et al., 2008). This travel strategy has also been adopted by tropical gibbons (Chivers, 1974; Bartlett, 2009; Raemaekers, 1978), with two groups of *H. lar* found to travel on average 1 330 m (range from 620 m to 1 930 m, *n*=12 months) and 1 160 m (range from 720 m to 1 660 m, *n*=12 months), they traveled short distance when dominantly feeding on leaves (Bartlett, 2009). However, variation in both time spent traveling and daily path lengths are more dramatic in northern gibbons (Fan & Jiang, 2008; Zhang et al., 2014). For example, the daily path length of *N. concolor* in Mt. Wuliang varies monthly from 629 m to 2 356 m (*n*=14 months) and daily from 300 m to 3 144 m (*n*=66 days, Fan & Jiang, 2008). The mean daily path length of *H. tianxing* in Mt. Gaoligong varies monthly from 508 m to 1 926 m (*n*=14 months) and daily from 345 m to 2 606 m (*n*=63 days) (Zhang et al., 2014). Furthermore, travel time can show seasonal differences. For example, the *H. tianxing* group in Mt. Gaoligong has been observed to travel (on average) 3.40 h in June but 1.27 h in February (Fan et al., 2013). Seasonal impact on travel time has also been reported for the two groups of *N. nasutus* in Bangliang (group G1: from 3.92 h in June to 0.52 h in March; group G4: 4.92 h in June to 1.37 h in March; Fan et al., 2012).

Both sleeping and resting behaviors in northern gibbons are strongly influenced by cold temperatures, with more time spent inactive (sleeping and resting) during the 24 h cycle in winter (Fan et al., 2012, 2013). In Nankang, Mt. Gaoligong, the *H. tianxing* group spent 20.12 h inactive in February, whereas the two groups of *N. nasutus* in Bangliang spent 20.23 h (December) and 19.69 h (January) inactive. Gibbons maintain an energy-conserving posture while sleeping or resting (sitting with hands clasping their legs: Bicca-Marques & Calegaro-Marques, 1998). Northern gibbons usually sit huddled together on tree tops in the sunshine during cold winter days, and even huddle and share sleeping sites on cold nights (Fan et al., unpublished data).

## SOCIAL SYSTEM OF NORTHERN GIBBONS

Gibbons usually live in small social groups with one adult pair and 2–3 offspring (Leighton, 1987). However, some northern gibbons live in stable polygynous groups with two breeding females (*N. hainanus*: Zhou et al., 2008; *N. concolor*: Fan & Jiang, 2010a; Guan et al., 2013; Huang et al., 2013; *N. nasutus*: Fan et al., 2010, 2015). Gibbon groups with more than one breeding female have also been reported in other species (*H. pileatus*: Srikosamatara & Brockelman, 1987; *Hoolock hoolock*: Ahsan, 1995; *H. lar*: Sommer & Reichard, 2000), however, none of these groups have remained stable for more than two years.

Gibbon groups with more than one adult female were first reported in *N. hainanus* in Delacour’s book *On the Indochinese gibbon* (Xu et al., 1983), and later in *N. concolor* from Mt. Wuliang by Haimoff et al. (1986, 1987). Sheeran (1993) did not observe any multi-adult female groups in Mt. Wuliang or Mt. Ailao; based on brief observations in the same areas, Bleisch & Chen (1991) reported that some *N. concolor* groups included two females who duetted with the adult male, though they argued that this could be a sub-adult daughter singing with her mother. For a clear demonstration of polygyny, close observation of groups with two or more females (with young sired by the same male) is required (Bleisch & Chen, 1991). Jiang et al. (1994a, 1999) suggested that the groups they observed were polygynous because the two females presented with offspring of the same body size. They thus proposed several models to explain the evolution of polygyny in *N. concolor*. First, the large home range occupied by *N. concolor* and *N. hainanus* might provide sufficient space for more individuals in one group. Second, mutual tolerance of females or “weak territoriality in females” might facilitate polygyny. Third, phylogeny might play a role in the evolution of polygyny in *Nomascus* gibbons. They also argued that *N. concolor* has a relatively large semi-pendulous scrotum (Groves, 1972), which should promote higher sperm production and, consequently, support a higher level of sexual activity. However, the above findings raise questions of why and how these gibbons occupy a large home range and females tolerate each other. These questions remained unanswered at the time as no gibbon groups had been habituated for detailed behavioral observation.

In 2006, Fan et al. (2006) reported that all five groups in a small subpopulation of *N. concolor* at Dazhaizi, Mt. Wuliang, included two adult females who gave birth or carried infants during the two-year research period. However, females in the same group bred at different times, perhaps to reduce reproductive competition. During the study, the researchers habituated one group and were the first to document the social behavior between two females living in the same group. They reported that the two females fed in the same tree, sang synchronously, groomed each other, and groomed and were groomed by the adult male, but never slept in the same place (Fan et al., 2006). They even shared meat when one female killed a flying squirrel (Fan & Jiang, 2009) and cooperated in evicting a “floating” (unmated) female from their territory (Fan et al., 2006). Further longitudinal observations showed that the two females repeatedly bred with the resident group male (Huang et al., 2013) and maintained similar spatial relationships to him (Fan & Jiang, 2010a). More recently, Guan et al. (2013) demonstrated that these groups consisted of two breeding females even after replacement of breeding males and females, and females actively cooperated in maintaining their social relationship, rather than co-existing merely through tolerance or avoidance.

The rediscovery of a *N. nasutus* population has provided further opportunity to study the evolution of polygyny in gibbons. This population lives in a degraded karst forest patch (Fan et al., 2011a), which is distinct from the habitat of any other gibbon population. Most groups in this population include two adult females. Fan et al. (2015) documented that *N. nasutus* benefits from living in larger groups because these groups can invade the home range of smaller gibbon groups and access more non-fig fruit, though larger groups also spend more time traveling. Female cooperation to evict unmated females has also been observed in this population but no group consisted of more than two breeding females, suggesting that territories defended by a male could not support a third female (Fan et al., 2015).

Long-term population monitoring can provide insight to better understand the sociality of northern gibbons. Although floating males in *N. concolor* populations have been observed regularly at Dazhaizi (Fan et al., unpublished data), females in the polygynous groups do not disperse and form new groups with these floating males. Two newly formed *N. nasutus* groups at Bangliang consisted of two adult females (Wei et al., 2017; Shao-Gan Wei, personal communication), though solitary males were observed frequently (Fan et al., 2010, 2015). Similar results have been reported from a small population of *N. hainanus*; although solitary males were observed living in the site, one of the two newly formed groups consisted of two females (Bryant et al., 2016; Deng et al., 2017). These results reject the hypotheses that females are forced to live in their natal groups by delayed dispersal or are forced to immigrate into monogamous groups because suitable habitat and mates are not available (Bleisch & Chen, 1991; Liu et al., 1989). Consequently, Fan et al. (2015) proposed that polygyny is an evolutionarily stable mating system in certain habitats and for some species.

**Figure 2:**
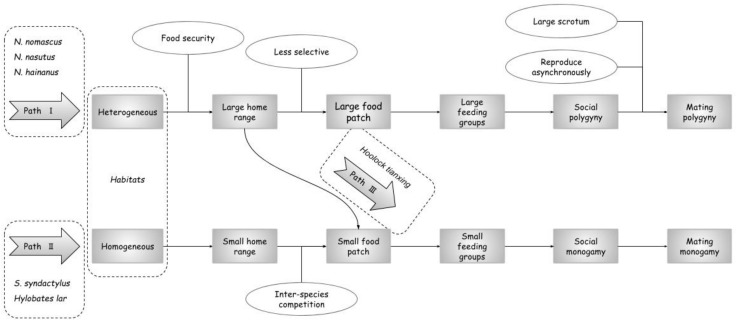
A revised model for the evolution of gibbon social and mating systems, integrating food distribution, patch size, gibbon digestibility and diet, inter-species food competition and reproductive behavior in the model

To secure their resource requirements, northern gibbons need to maintain a large territory in their heterogeneous habitats (Liu et al., 1989; Fan & Jiang, 2008, 2010b; Zhang et al., 2014). Within each territory, important food species occur in large patches, which can provide adequate food for two breeding females and their offspring, but perhaps not enough for three females (Fan & Jiang, 2008; Fan et al., 2015). The ability to digest substantial amounts of leaves in response to fruit scarcity (Fan et al., 2009a, 2012) enables these gibbons to minimize feeding competition within groups when fruit is not available and forage in large polygynous groups. Adult *Nomascus* males have a large scrotum and females in the same group breed at different times (*N. hainanus*: Zhou et al., 2008; *N. concolor*: Huang et al., 2013), which both reduce reproductive competition and enables polygyny (Path I in Figure 2). Southern gibbons live in more homogenous habitats (e.g., *S. syndactylus*: Chivers, 1974; *H. lar*: Bartlett, 2009; Brockelman et al., 2014), with smaller home ranges (Bartlett, 2011). Although they utilize large fruit trees (Bartlett, 2009), intensive interspecific competition depletes food resources, which may promote within-group competition (Marshall et al., 2009) (Path II in Figure 2). That does not mean food patch size in the tropical gibbon habitat is physically smaller than that for northern gibbons, but that on average, food availability is less.

Males benefit from forming polygynous groups through increased mating opportunities (*N. hainanus*: Zhou et al., 2008; *N. concolor*: Huang et al., 2013; *N. nasutus*: Fan et al., 2015). The cooperative female relationships documented above (*N. concolor*: Fan et al., 2006; Fan & Jiang, 2010a; Guan et al., 2013; *N. nasutus*: Fan et al., 2015) suggest that females in polygynous groups also benefit from larger group sizes. The potential benefits to females include resource access from successful intergroup encounters (Fan & Jiang, 2010a; Fan et al., 2015). Resident females in the same group appear to produce similar-aged offspring. After maturing, these offspring can disperse together, which might increase their survival rate and probability of establishing their own territory (Fan & Jiang, 2010a; Fan et al., 2015). In addition, females might benefit from improved inclusive fitness if females born within the same group disperse together (Fan et al., 2015). Even if females do not obtain any direct or indirect benefits from polygyny, bi-female groups could form if the cost of evicting the second female exceeds the cost of tolerance (Fan & Jiang, 2010a; Fan et al., 2015).

We need to note that *H. tianxing* living in northern montane forest shares many similarities in ecology with the three *Nomascus* species (reviewed above), but no stable group of more than one adult breeding female has been reported. A possible reason is that food patch size in the habitat of *H. tianxing* is smaller than that of *Nomascus* species (Path III in Figure 2). Given that stable polygynous groups have only been reported from *Nomascus* species living in different habitats, phylogeny may also play an important role in the evolution of polygyny in gibbons (Bryant et al., 2015; Di Fiore & Rendall, 1994; Jiang et al., 1999).

## FUTURE RESEARCH DIRECTIONS

### Standardized methods

Comparative research using the same or similar methods will be extremely useful for understanding gibbon ecology and social system evolution. First, the same methods should be applied to quantify forest structure, plant abundance and distribution, and food availability (Vogel & Janson, 2011). Second, the same or similar behavioral definitions and methodologies should be used to record behavior. For example, several researchers distinguished foraging as “*the group moved slowly, spread out over a broad front, feeding briefly from many of the trees as they passed through them*” from a feeding bout when all group members fed in the same tree at the same time. Kappeler (1981) and Ahsan (2001) recorded foraging, but other researchers did not separate foraging from feeding (Bartlett, 2009; Fan et al., 2008, 2012, 2013; McConkey & Brockelman, 2011). Palombit (1997) used focal animal observations to record behavior, whereas other researchers have used scan sampling (Ahsan, 2001; Chivers, 1974; Fan et al., 2008, 2012, 2013; Gittins, 1982; Lappan, 2007a). Even for researchers using scan sampling, scan intervals often differ (e.g., 5 min: Bartlett, 2009; Fan et al., 2008, 2012, 2013; 10 min: Ahsan, 2001; Gittins, 1982; 30 min: McConkey & Brockelman, 2011). Furthermore, comparisons would be strengthened if researchers used the same method to analyze data. Although most researchers present diet and time budget results as percentages (Ahsan, 2001; Bartlett, 2009; Fan et al., 2008, 2009a; Gittins, 1982; McConkey & Brockelman, 2011), Fan et al. (2012, 2013) used time devoted to different activities and food categories because active time showed considerable disparity as a consequence of variations in day length at high latitude areas. It would strengthen the ability to compare results if all authors published their data as both percentages and actual times for both diet and time budgets. Finally, altitudinal range must be considered when calculating home range size and daily travel length because both variables can be impacted at steep study sites (Monterroso et al., 2013).

### How do northern gibbons defend their large home range?

Gibbons are territorial and regularly and aggressively defend their home range, which is thought to enable exclusive access to a feeding territory (Bartlett, 2009; Brockelman, 2009). Northern gibbons occupy home ranges several times larger than those of tropical gibbons (*N. concolor*: Fan & Jiang, 2008; *N. nasutus*: Fei et al., 2012). However, they do not travel any longer than gibbons that occupy smaller home ranges. Gibbons use their loud morning singing to defend their territories. However, northern gibbons do not increase singing to defend their larger home ranges (Fan et al., 2009b; Yin et al., 2016). In fact, hoolock gibbon groups appear to decrease their singing in areas with low group density (Yin et al., 2016). Therefore, the way in which northern gibbons defend their large territories still requires further investigation.

### Social relationship in groups with different social systems

Intragroup social behavior in gibbons has received little attention (Bartlett, 2011). From the limited information available, males have been observed grooming females more often than the reverse in the monogamous groups of *S. syndactylus* (Chivers, 1974; Palombit, 1996), *H. lar* (Palombit, 1996), and *H. hoolock* (Ahsan, 2001). Guan et al. (2013) studied the grooming patterns of two polygynous groups of *N. concolor* and found that females groomed males more often than the reverse, a pattern also observed in other polygynous primates (Ahsan, 2001). Studying intragroup social relationships in populations with different social systems will help to better understand the evolution and maintenance of social systems in gibbons.

### Are northern gibbons adapted to digesting leaves?

Northern gibbons eat more leaves than do tropical gibbons (but see *S. syndactylus* in Bartlett, 2011, Table 17.2). However, how they resolve the chemical and mechanical challenges associated with leaf eating (Lambert, 1998), especially during cold winters with reduced or no fruit, remains unclear. Thus, comparative research on their digestion system, including their microbiome, will help clarify their ecology and social systems.

### Kinship among females

Female kinship may be a factor affecting social relationships (Clutton-Brock & Lukas, 2012). Females living in polygynous groups may benefit through inclusive fitness if they are kin, and is highly possible due to the previous observation of two *N. nasutus* females dispersing together (Fan et al., 2010). However, as it is difficult to observe group formation or female displacement in the wild (Brockelman et al., 1998), the use of non-invasive genetic methods may help determine kin relationships among females (Lappan, 2007b).

### How do northern gibbons find food within their large home ranges?

Do northern gibbons use spatial memory to find food resources in the same manner as tropical gibbons (Brockelman et al., 2014)? In northern habitats, gibbons appear to prefer ripe fruit pulp (Fan et al., 2009a, 2013). Scarce but predictable food resources led to the development of the “ecological intelligence hypothesis” (Milton et al., 1981), that is, primates, such as great apes, which rely on ephemeral and scattered fruits need larger ranges and have evolved advanced cognitive capacities that facilitate optimal food finding strategies. Does the “ecological intelligence hypothesis” explain the foraging behavior of gibbons? Long-term comparative research across gibbon species and between gibbons and large-bodied apes will help elucidate northern gibbon foraging behavior in their larger home ranges.

### Comparison between foraging behavior

Given the different interspecies food competition at different sites, tree diameter at breast height (DBH) or crown size may not be accurate indicators of food availability. We highly recommend using feeding party size, feeding bout length, and number of feeding patches visited as indices to represent food availability, especially as gibbons often deplete their food patches. However, this prerequisite needs careful investigation. If these indices are validated, then detailed comparison among the foraging behaviors of gibbons living in different social systems can be made.

### Life history and cultural behavior

Gibbons born in polygynous groups may develop better social skills for maintaining relationships due to their interactions with more social partners while growing up. Therefore, ontogeny may also play a role in the maintenance of polygyny. Female gorillas familiar from immaturity usually exhibit friendly relationships in adulthood (Harcourt, 1979). Studying the development of social relationships in gibbons is difficult because of their long lifespans, and thus longitudinal data is required to answer the above question. Only with long-term study and the accumulation of data can we reveal unusual observations, such as “dance” (Fan et al., 2016) and “juggling” behaviors (Deng & Zhou, 2016). Current evidence implies that there is a great deal more to learn about gibbon species, and a wide variety of behavior remains to be explored in future studies.
